# Linkage of Depression with Elder Abuse among Institutionalized Older Persons in Kathmandu Valley, Nepal

**DOI:** 10.1155/2021/5546623

**Published:** 2021-04-28

**Authors:** Maginsh Dahal, Smriti Dhakal, Sudip Khanal, Kushalata Baral, Saroj Mahaseth

**Affiliations:** ^1^Department of Public Health, Asian College for Advance Studies, Purbanchal University, Lalitpur, Nepal; ^2^Department of Public Health, Nobel College, Pokhara University, Sinamangal, Kathmandu, Nepal

## Abstract

**Background:**

To identify the association between elderly abuse and depression among elderly living in old age homes of Kathmandu Valley, Nepal.

**Materials and Methods:**

A cross-sectional study was carried out in 5 old age homes of Kathmandu Valley, Nepal. The Geriatric Mistreatment Scale and the Geriatric Depression Scale were used to collect information from 220 elderly aged 65 or above, and face-to-face interviews were conducted. Bivariate and multivariate logistic regression analyses were carried out to identify the association between elderly abuse and geriatric depression.

**Results:**

Among the different types of abuse analyzed, the multivariate analysis showed that neglect (AOR = 2.995; CI: 1.249-7.181) and economical abuse (AOR = 4.728, CI: 1.836-12.173) were significantly associated with increased risk of geriatric depression. Furthermore, the study identified that future saving and absence of chronic disease significantly reduced the risk of psychological abuse, neglect, and geriatric depression.

**Conclusions:**

Neglect and economical abuse are a predictor of geriatric depression. Efforts should be directed to increase awareness about the different forms of abuse among the primary caregivers of the elderly. Counseling services and support programs should be introduced in old age homes to address the high burden of geriatric depression.

## 1. Introduction

The world's population is ageing in an unprecedented manner. There is a rapid increase in the number of elderly population worldwide and it has been ascribed as the most powerful demographic force today [1]. According to the World Health Organization, the elderly population refers to individuals who are 65 years of age or older [2]. This period is often accompanied by chronic disease, functional impairments, and decreased social interaction, which makes the elderly more dependent on caregivers and puts them at a greater risk of being abused [3].

Elderly abuse is defined as “a single or repeated act, or lack of appropriate action, occurring within any relationship where there is an expectation of trust which causes harm or distress to an older person.” It can be either physical, psychological, economic, sexual abuse, or neglect. Exposure to any form of elderly abuse gravely affects the physical and mental health of an elderly, and it can lead to long-lasting psychological consequences, including depression [3]. Depression is a mental health condition characterized by sadness, loss of interest or pleasure, feelings of guilt or low self-worth, disturbed sleep or appetite, feelings of tiredness, and poor concentration [4]. Depression during old age can substantially reduce the quality of life and it is increasingly being recognized as a major public health concern [5].

Globally, the WHO estimates that 15.7% of older people living in the community setting suffer from elderly abuse [6, 7]. In the institutional setting, the numbers are much higher. It was found that 64.2% of the staff working in institutional settings had perpetrated a type of abuse against the elderly [6, 8]. However, these data represent only the tip of the iceberg as only 4% of the elderly abuse cases get reported [6]. Furthermore, depression is identified as the leading cause of disability worldwide. The WHO data shows that depression peaks during older adulthood, affecting 5.5% of males and 7.5% of females worldwide [8]. Similarly, a study conducted in India shows that depressive symptoms were quite high among the elderly in both the setups. Special attention should be given toward health checkups of depressed persons in the OAHs (old age homes) and improvement of family ties among depressed persons of the community [9].

In Nepal, there is no national prevalence data on elderly abuse or depression. A study conducted by the Geriatric Center Nepal on the reported cases of elderly abuse showed that 49 out of 77 districts had reported cases of elderly abuse, with the highest being reported from Kathmandu and Morang district [10]. Strikingly, the small-scale studies conducted here show appallingly high numbers. Studies show that 50.3% of the elderly living in the community setting [11] and 58% of the elderly living in institutional settings [12] are victims of elderly abuse. Likewise, the prevalence of depressive symptoms ranged from 25.5% to 60.6% in community settings, 17.3% to 89.1% in care facilities, and 53.2% to 57.1% in hospital settings [13, 14].

These data, although from small-scale studies, show that both elderly abuse and depression are widely prevalent in Nepalese society. However, there is a paucity of research conducted on these topics because of a multitude of reasons. To begin with, ageing was not considered a major public health concern in developing and underdeveloped nations for a very long time and Nepal was no exception [15]. Furthermore, Nepal is a country where elderly abuse and depression are still considered taboo topics and the stigma around them is so strong that victims or patients would rather not seek help than talk about it. As a result, there has been very little open discussion on these issues [10, 16]. Moreover, elderly abuse and depression are not just a national concern.

Global data shows that every country in the world is experiencing growth in the size and proportion of elderly in their population and population ageing has been the fastest in the Eastern and South-Eastern Asia [17]. It is projected that the proportion of older adults will increase by more than twofold worldwide by 2050 [17]. Furthermore, elderly abuse and depression are prevalent in every country, with their burden high in institutional settings, and are a major public health threat for geriatric health throughout the globe [8, 18]. Despite this, there is a lack of research studies on elderly abuse and depression in institutional settings, especially in low- and middle-income countries [8, 19]. Limited studies are available on elderly abuse and geriatric depression and in the Nepalese context; none has yet captured the association between these two variables. This makes it imperative that we enrich the available global as well as national evidence for sound policy decisions on geriatric health. Hence, through our study, we aim to examine the association between elderly abuse and depression among elderly living in the old-age homes of Kathmandu Valley of Nepal.

## 2. Materials and Methods

A descriptive cross-sectional quantitative study was carried in 5 randomly selected old-age homes out of 11 old age homes of Kathmandu Valley of Nepal. Kathmandu Valley was chosen as the study area because of the high report of elderly abuse in the area [10] and the rapid increase in the number of old age homes [12, 20]. The sampling frame for the study was the total number of elderly living in the old age homes in Kathmandu Valley, which was identified through the Social Welfare Council of Nepal. Elderly aged 65 years or above were considered for the study. The sample size was first calculated for the infinite population and then for the finite population using the formulas below:

(1) $$\begin{array}{c} n_{0}={\left(\frac{Z_{\alpha }}{E}\right)}^{2}PQ, \end{array}$$

where *n*_0_ = sample size for infinite population, *α* = 5%(level of significance), *Z*_*α*_ = *Z*_0.05_ = 1.96, *E* = 0.05 (error), and *P* = 0.578.

Using the formula, the sample sizes for the infinite population were found to be 374.32~375 and 374.81~375, respectively. The sample size was then calculated for the finite population using the following formula:

(2) $$\begin{array}{c} n=\frac{n_{0}}{\left(1+\left(n_{0}/N\right)\right)}, \end{array}$$

where *n* = sample size for finite population, *n*_0_ = sample size for infinite population, and *N* = 500 (estimated number of elderly living in old age homes obtained through the Social Welfare Council):

(3) $$\begin{array}{c} n=\frac{375}{\left(1+\left(375/500\right)\right)},\\ n=214.28\sim 215 \end{array}$$

To avoid any missing data, the sample size taken for this study was 220. Simple random sampling using the lottery method was carried out to select the old age homes to be studied from the list of the total old age homes in Kathmandu Valley of Nepal, and the census was carried out in the selected old age homes. The inclusion criteria for the study were kept as broad as possible—only the elderly who could not speak or listen, who were reported to be mentally unfit for the study by the authorities at the old age homes and who refused to participate were excluded.

The study was approved by the Institutional Review Committee (IRC) of Nobel College, Sinamangal. Privacy and confidentiality of the data were ensured to each participant before collecting any information. They were also assured that the collected information will be used for research purposes only and their personal identity will not be revealed. The participants were provided full information about the research before taking verbal consent, and they were allowed to leave the study whenever they wanted. Verbal consent was taken from the participants instead of written consent because studies showed that the elderly feared that their signs or thumbprints might be misused [12].

The data was collected through face-to-face interviews by the researcher themselves after pretesting with 20 participants, who were not further used in the study, using the Geriatric Mistreatment Scale (GMS) [21] and the Geriatric Depression Scale (GDS) [22] after taking formal permission to use/modify tools. The tools were then translated to the local language for validity [21]. The interview was approximately for 17–18 minutes each. The study was conducted within six months. The GDS is a 15-item scale with dichotomous responses and moderate internal consistency (Cronbach's *α* = 0.82) [22]. A score of >5 was considered suggestive of depression and a score of ≥10 was considered indicative of depression [23]. The collected data was analyzed using Statistical Package for the Social Sciences (SPSS) version 20.0. Descriptive statistics like mean and standard deviation were calculated for quantitative variables, and frequency and percentage were calculated for categorical variables. The measures of central tendency were rounded off to one decimal place and measures of dispersion were rounded off to two decimal places. Chi-square test of independence was performed to identify the association. Bivariate and multivariate logistic regression was carried out wherever applicable. For all tests, two-sided *α* of 0.05 was considered statistically significant.

## 3. Results

In Table 1, among a total of 220 respondents, the mean and SD age of the respondents was 79.87 ± 8.160 years and 79.1% of the total respondents were female. More than half of the respondents (61.4%) were Hindu and 49.1% belong to the ethnic group Janajati and Dalits. Regarding marital status, 67.7% of the respondents did not have a life partner because they were divorced, separated, widowed, or unmarried and 63.2% the elderly were living in a nuclear family before living in old age home. Nearly half of them (49.1%) could not read or write. Similarly, 75.0% of the respondents did not have any future savings and the majority of them were suffering from chronic diseases, with the top contender being cardiovascular disease (52.1%). The presence of different types of abuse among the elderly was identified, and it was found that the majority were victims of psychological abuse (78.6%), followed by neglect (57.3%) and economic abuse (57.3%).

Table 2 depicts the results of the binary regression analysis performed to check the association between sociodemographic variables and the types of abuse. The findings showed that elderly who had future savings were 50.7% less likely to suffer from psychological abuse compared to those who did not have future savings (AOR = 0.493, CI: 0.256-0.951). Elderly who had no chronic disease were 65.5% less likely to experience psychological abuse compared to their counterparts (AOR = 0.345, CI: 0.178-0.667). Furthermore, the study identified that Christians were 3.394 times more likely to experience neglect than Hindus (AOR = 3.394, CI: 1.611-7.152). The elderly who had future savings were 67.2% less likely to experience neglect compared to those who did not have future savings (AOR = 0.328, CI: 0.145-0.741). Respondents with no chronic disease were 74.8% less likely to experience neglect compared to those who had chronic disease (AOR = 0.252, CI: 0.111-0.574). With regard to economic abuse, the study found that elderly who lived in nuclear families before living in old age homes were two times more likely to experience economic abuse than those who lived in joint or extended families (AOR = 1.955, CI: 1.051-3.638). Likewise, the elderly who suffered from cardiovascular diseases were two times more likely to experience economic abuse compared to their counterparts (AOR = 1.971, CI: 1.099-3.535).

Table 3 shows the association between different types of abuse and the status of depression. It was identified that the major abuse types like physical abuse, psychological abuse, neglect, and economic abuse had a statistically significant association with the depression status of the elderly with a *p* value < 0.001.

Table 4 depicts the crude and adjusted odds ratio of variables that were found to be significantly associated with the status of depression. Our study identified that Buddhist respondents were 67.3% less likely to be suggestive of depression (AOR = 0.327, CI: 0.136-0.787) compared to the Hindu respondents. Elderly who had future savings were 73.2% less likely to be suggestive of depression (AOR = 0.268, 0.125-0.574) and 75.7% less likely to be indicative of depression (AOR = 0.243, CI: 0.077-0.762) compared to their counterparts. The respondents who had no chronic disease were 65.0% less likely to be suggestive of depression (AOR = 0.350, CI: 0.170-0.721) and 77.2% less likely to be indicative of depression (AOR = 0.228; CI: 0.072-0.720) compared to those who had chronic diseases.

Each abuse type was seen to be highly significant for the depression status in the bivariate analysis; however, after adjusting the association for other abuse types, only a few remained significant. It was found that elderly who were neglected were three times more likely to be suggestive of depression (AOR = 2.995; CI: 1.249-7.181) compared to those who were not neglected. Furthermore, elderly who suffered from economic abuse were nearly five times more likely to be indicative of depression (AOR = 4.728, CI: 1.836-12.173) compared to their counterparts.

## 4. Discussion

The findings of our study were compared mostly with studies conducted in Asia, and we ensured that all depression-related articles used the Geriatric Depression Scale so that the comparison could be done on fairly similar grounds.

This study showed that the most common form of abuse among respondents was psychological abuse (78.6%). A meta-analysis conducted on elderly abuse in an institutional setting showed that majority, i.e., 33.4% of the elderly suffered from psychological abuse [8]. In India, a study showed that psychological abuse affected 50% of the respondents [24]. These studies support our results by indicating psychological abuse as the most common form of elderly abuse. However, another study conducted in Nepal showed that majority, i.e., 47.4%, of the elderly were suffering from neglect [12]. This contrast is explainable as neglect was identified to be the second most common form of abuse in our study, affecting nearly 58% of the respondents, and given our large sample size compared to the study above [12], the contrast might simply be because we screened more populations in a vulnerable setting.

Our study showed that three-fourths of the elderly living in old age homes had no future savings. This finding is congruent with another study conducted in old age homes of Kathmandu where 68% of the elderly did not have any financial support [25]. However, a study conducted in a community setting in Nepal showed that majority, i.e., 61.4%, of the elderly had future savings [26]. These contradictory findings might be because of the difference in the research setting as elderly living in old age homes are more likely to have a history of economic abuse which deprives them of their future savings. Furthermore, this study showed that most of the elderly were suffering from one or more chronic health conditions which is similar to the findings of another Nepal-based study where 72% of the older adults living in the old age home in Kathmandu were suffering from chronic diseases [25].

This study showed that having future savings significantly reduced the risk of psychological abuse and neglect among the elderly. According to a study conducted in Nepal, there was no significant association between future savings and elderly abuse (Pearson correlation coefficient: 0.101) [26]. In India, a study conducted showed no significant association between the income status of the elderly and abuse (*χ*^2^ = 0.13, *p* value: 0.710) [27]. These studies are in contrast to our study findings. This is likely because the articles referred were conducted in community settings where future saving was found to be better distributed among the respondents.

In our study, it was found that the elderly who had no chronic disease were at less risk of psychological abuse and neglect compared to their counterparts. A study conducted in Nepal showed that elderly who were taking medications for morbidity were two times more likely to suffer from psychological abuse than their counterparts (AOR: 1.82, 95% CI 1.01-2.83) [28]. In India, a study showed that having no morbidity had a protective effect against suffering from neglect (OR: 0.67, *p* value: <0.1) or any kind of abuse in general (OR: 0.47, *p* value: <0.01) [29]. These studies support our results by showing that the elderly who do not have any morbidity are at less risk of abuse. A possible explanation here might be that the elderly who do not have chronic diseases do not require extra care and this might reduce the likelihood of them feeling that they are not being treated properly by their caregivers.

Results of the multinomial regression analysis performed in our study showed that having future savings significantly decreased the risk of depression among the elderly. A study conducted in India showed a strong relationship between financial dependency and geriatric depression (*χ*^2^ = 24.8, *p* value: <0.001) [30]. Another study conducted in India showed that the elderly who were financially dependent on others were 2.49 times more likely to develop depression compared to their counterparts (UOR = 2.49, 95% CI 1.81-3.42, *p* value: <0.001) [31]. These results support our findings and indicate that having a future saving reduces the risk of geriatric depression. A possible explanation behind this might be that future saving is likely to reduce the worry of financial security among older adults and the sense of financial independence that the elderly gain might reduce their anxiety and make them less vulnerable to depression.

In our study, the absence of chronic disease was found to significantly reduce the risk of geriatric depression. A study conducted in Nepal showed that elderly who had chronic diseases were two times more likely to suffer from depression compared to their counterparts (AOR: 1.8, 95% CI 1.2-2.8, *p* value: 0.012) [32]. Another Nepal-based study showed that elderly who had multiple chronic diseases were 1.67 times more likely to suffer from depression than their counterparts (AOR: 1.67, 95% CI 1.09-2.55, *p* value: 0.018) [33]. A national survey in Sri Lanka showed that elderly who had chronic diseases were more likely to develop depression symptoms (AOR: 1.11, 95% CI 0.69-1.76) [34]. These studies match the findings of our research and further support that the absence of chronic disease can reduce depression among elderly. This might be because the elderly who have no chronic health conditions do not have to go through the physical burden of the illness as well as through the mental health burden that arises as a result of the need for more tangible and intangible care for chronic diseases.

Our study checked the association between abuse types and depression. In the bivariate analysis, a highly significant association was seen between each abuse type and depression, but when the association between a particular type of abuse and depression was adjusted for other abuse types, no significant association was seen in some. Considering this is essential for our study as we move forward because most of the articles we referred show highly significant associations between abuse types and depression, but they have not taken into account how this association is affected by the presence of other types of abuse. This might hint towards the complex relationship between elderly abuse and depression and the paucity of research conducted to check their association, thus warranting the need for more research in this area.

In the bivariate regression analysis, our study found that physical abuse and psychological abuse significantly increased the risk of geriatric depression. However, after adjusting for other types of abuse, no significant association was seen. A study conducted in Brazil showed that elderly who were mistreated physically (AOR: 1.754, 95% CI 1.076-2.861, *p* value: ≤0.01) and psychologically (AOR: 2.252, 95% CI 1.351-3.754, *p* value ≤0.01) during their early years were more likely to develop geriatric depression [35]. Another study identified that individuals who were exposed to psychological abuse were at a higher risk of developing geriatric depression [36]. These studies are in contrast to the findings of our study. This is likely because the studies referred did not adjust the identified association with all four types of abuse included in our study. This explanation can be backed up by another research conducted in Portugal where physical abuse was found to be significantly associated with depression in the initial bivariate analysis, but no association was seen when analyzed regarding financial abuse (PR: 1.19 95% CI 0.58-1.44, *p* value: 0.625) [37].

This study showed that elderly who were neglected were three times more likely to be suggestive of depression compared to their counterparts. A study conducted in Brazil studied two different domains of neglect and it was found that both physical neglect (AOR: 1.912, 95% CI 1.179-3.103, *p* value: ≤0.01) and emotional neglect (AOR: 2.822, 95% CI 1.698-4.692, *p* value: ≤0.001) significantly increased the risk of geriatric depression [35]. Another study identified that individuals who were exposed to neglect were at a higher risk of developing geriatric depression [36]. Further, a study conducted in the US showed that a higher level of neglect during early life was related to higher depressive symptoms in old age (*β* = 2.79, *p* ≤ 0.05) [38]. These findings support the conclusion of our study and it identifies neglect as a predictor of geriatric depression. A possible explanation behind this might be that neglect at any point in life can deprive an individual of much-needed love, care, attention, and emotional support, thus making them despondent and at risk of depression.

In our study, it was identified that the elderly who were economically abused were at a higher risk of depression compared to their counterparts. A study conducted in Iran showed a significant association between financial abuse and depression (*p* = 0.036, *β* = 0.117) [39]. The findings from a book on elder mistreatment stated that financial abuse can lead to depression among the elderly [40]. These findings are consistent with our study results. This is likely because losing an asset through deceit can cause loss of trust and a constant sense of financial insecurity among individuals, which in the long run might aggravate and result in geriatric depression.

## 5. Conclusion

This study identified that a significant association exists between elderly abuse and depression. Among the different types of abuse studied, it was identified that neglect and economic abuse among the elderly played a major role in the development of geriatric depression. In addition, it was identified that future savings and the absence of chronic disease played a protective role against psychological abuse, neglect, and depression among elderly.

## 6. Limitations

Our study is not free from limitations. The sample size used in our study was limited, which may prevent us from making broad generalizations. Furthermore, the study was conducted through face-to-face interviews, which might create a possibility of asking bias.

## Figures and Tables

**Table 1:** Demographic characteristics of the respondents (*n* = 220).

Characteristics	*n* (%)
Age mean age ± SD (in years)	79.9 ± 8.16
Gender	
Male	46 (20.9)
Female	174 (79.1)
Religion	
Hindu	135 (61.4)
Buddhist	40 (18.2)
Christian	45 (20.5)
Ethnicity	
Brahmin	83 (37.7)
Kshetris	29 (13.2)
Janajati and Dalits	108 (49.1)
Marital status	
Married	71 (32.3)
Divorced/separated/widowed	149 (67.7)
Family type	
Nuclear	139 (63.2)
Joint and extended	81 (36.8)
Literacy	
Cannot read and write	108 (49.1)
Can read and write	49 (22.3)
Primary level	39 (17.7)
Secondary level and above	24 (10.9)
Future savings	
Yes	55 (25.0)
No	165 (75.0)
Chronic diseases (multiple responses)	
Diabetes	45 (21.1)
CVD	111 (52.1)
Arthritis	61 (28.6)
Others;	6 (2.8)
Do not have chronic diseases	58 (27.2)
Types of abuse (multiple responses)	
Physical abuse	48 (36.6)
Psychological abuse	103 (78.6)
Neglect	75 (57.3)
Economical abuse	75 (57.3)
Sexual abuse	10 (7.6)

**Table 2:** Binary regression analysis of factors associated with types of abuse.

Characteristics	UOR (95% CI)	AOR (95% CI)	*p* value
Psychological abuse			
Future savings (no)	0.458 (0.241-0.869)	0.493 (0.256-0.951)	0.035^∗^
Do not have chronic disease (no)	0.328 (0.l71-0.631)	0.345 (0.178-0.667)	0.002^∗^
Neglect			
Religion (Hindu)			
Buddhist	1.275 (0.595-2.733)	1.204 (0.544-2.666)	0.647
Christian	3.311 (1.646-6.661)	3.394 (1.611-7.152)	0.001^∗^
Future savings (no)	0.293 (0.135-0.640)	0.328 (0.145-0.741)	0.007^∗^
Do not have chronic disease (no)	0.267 (0.123-0.581)	0.252 (0.111-0.574)	0.001^∗^
Economic abuse			
Family type (joint and extended)	1.997 (1.086-3.671)	1.955 (1.051-3.638)	0.034^∗^
CVD (no)	2.124 (1.200-3.761)	1.971 (1.099-3.535)	0.023^∗^
Arthritis (no)	1.836 (1.000-3.374)	1.663 (0.889-3.110)	0.111

Characteristics mentioned in brackets represent the reference category. UOR: unadjusted odds ratio; AOR: adjusted odds ratio. ^∗^Statistical significance.

**Table 3:** Association between elderly abuse and depression.

Types of abuse	Depression status			*p* value
	No (%)	Suggestive (%)	Indicative (%)	
Physical abuse				
Yes	9 (8.9)	26 (29.9)	13 (40.6)	<0.001^∗^
No	92 (91.1)	61 (70.1)	19 (59.4)	
Psychological abuse				
Yes	28 (27.7)	50 (57.5)	25 (78.1)	<0.001^∗^
No	73 (72.3)	37 (42.5)	7 (21.9)	
Neglect				
Yes	15 (14.9)	41 (47.1)	19 (59.4)	<0.001^∗^
No	86 (85.1)	46 (52.9)	13 (40.6)	
Economical abuse				
Yes	19 (18.8)	35 (40.2)	21 (65.6)	<0.001^∗^
No	82 (81.2)	52 (59.8)	11 (34.4)	
Sexual abuse				
Yes	1 (1.0)	5 (5.7)	4 (12.5)	NA
No	100 (99.0)	82 (94.3)	28 (87.5)	

**Table 4:** Multinomial regression analysis of factors associated with depression.

Characteristics	Depression status (no)					
	Suggestive of depression			Indicative of depression		
	UOR (95% CI)	AOR (95% CI)	*p* value	UOR (95% CI)	AOR (95% CI)	*p* value
Religion (Hindu)						
Buddhist	0.397 (0.171-0.922)	0.327 (0.136-0.787)	0.013^∗^	0.628 (0.212-1.860)	0.505 (0.164-1.555)	0.234
Christian	2.120 (0.984-4.565)	1.920 (0.856-4.302)	0.113	2.008 (0.724-5.567)	1.848 (0.640-5.341)	0.256
Future savings (no)	0.254 (0.123-0.527)	0.268 (0.125-0.574)	0.001^∗^	0.227 (0.074-0.697)	0.243 (0.077-0.762)	0.015^∗^
Do not have chronic diseases (no)	0.374 (0.190-0.734)	0.350 (0.170-0.721)	0.004^∗^	0.237 (0.077-0.728)	0.228 (0.072-0.720)	0.012^∗^
Physical abuse (no)	4.357 (1.911-9.934	1.957 (0.752-5.089)	0.169	6.994 (2.617-18.691)	1.847 (0.581-5.871)	0.299
Psychological abuse (no)	3.523 (1.917-6.475)	1.321 (0.579-3.014)	0.509	9.311 (3.621-23.946)	3.012 (0.916-9.901)	0.069
Neglect (no)	5.110(2.560-10.202)	2.995 (1.249-7.181)	0.014^∗^	8.379 (3.429-20.478)	2.771 (0.929-8.261)	0.068
Economical abuse (no)	2.905 (1.505-5.608)	1.942 (0.953-3.956)	0.068	8.239 (3.404-19.940)	4.728 (1.836-12.173)	0.001^∗^

Characteristics mentioned in brackets represent the reference category. UOR: unadjusted odds ratio; AOR: adjusted odds ratio. ^∗^Statistical significance.

## Data Availability

This manuscript's datasets supporting this research will not be shared publicly and are available from the corresponding author upon reasonable request.

## References

[1] World Health Organization & US National Institute of Ageing (2011). *Global health and ageing*.

[2] World Health Organization (2008). *Men, Ageing and Health: Achieving Health across the Life Span*.

[3] Krug E., Dahlberg L., Mercy J. (2002). *World Report on Violence and Health; Chapter 5: Abuse of the Elderly*.

[4] World Health Organization (2017). *Depression and Other Common Mental Disorders: Global Health Estimates*.

[5] Cheruvu V. K., Chiyaka E. T. (2019). Prevalence of depressive symptoms among older adults who reported medical cost as a barrier to seeking health care: findings from a nationally representative sample. *BMC Geriatrics*.

[6] World Health Organization (2020). *Elder abuse*.

[7] Yon Y., Mikton C. R., Gassoumis Z. D., Wilber K. H. (2017). Elder abuse prevalence in community settings: a systematic review and meta-analysis. *The Lancet Global Health*.

[8] Yon Y., Ramiro-Gonzalez M., Mikton C. R., Huber M., Sethi D. (2019). The prevalence of elder abuse in institutional settings: a systematic review and meta-analysis. *European Journal of Public Health*.

[9] Zalavadiya D. D., Banerjee A., Sheth A. M., Rangoonwala M., Mitra A., Kadri A. M. (2017). A comparative study of depression and associated risk factors among elderly inmates of old age homes and community of Rajkot: a Gujarati version of the geriatric depression scale-short form (GDS-G). *Indian Journal of Community Medicine: Official Publication of Indian Association of Preventive & Social Medicine*.

[10] Geriatric Center Nepal (2010). A baseline study on reported cases of elder abuse in Nepali press.

[11] Chalise H., Paudel B. (2020). Elderly abuse among community-living older adults of least developed country-Nepal. *Archives of Physical Medicine and Rehabilitation*.

[12] Rai S., Khanal P., Chalise H. (2018). Elderly abuse experienced by older adults prior to living in old age homes in Kathmandu. *Journal of Gerontology and Geriatric Research*.

[13] Gautam P., Dahal M., Ghimire H. (2021). Depression among adolescents of rural Nepal: a community-based study. *Depression Research and Treatment*.

[14] Thapa D., Visentin D., Kornhaber R., Cleary M. (2018). Prevalence of mental disorders among older people in Nepal: a systematic review. *Kathmandu University Medical Journal*.

[15] Shrestha L. B. (2000). Population aging in developing countries. *Health Affairs*.

[16] Rijal A. (2018). Mental health situation in Nepal and priorities for interventions. *Health Prospect: Journal of Public Health*.

[17] United Nations (2019). *World Population Ageing 2019*.

[18] Ishveen C., Chopra A. (2014). Depression and Aging – A Public Health Concern.

[19] Pilania M., Yadav V., Bairwa M. (2019). Prevalence of depression among the elderly (60 years and above) population in India, 1997–2016: a systematic review and meta-analysis. *BMC Public Health*.

[20] Mishra S., Chalise H. (2019). Health status of elderly living in Briddaashram (old age home). *International Journal of Public Health and Safety*.

[21] Giraldo-Rodríguez L., Rosas-Carrasco O. (2013). Development and psychometric properties of the geriatric mistreatment scale. *Geriatrics & Gerontology International*.

[22] Sheikh J. I., Yesavage J. A. (1986). 9/Geriatric Depression Scale (GDS). *Clinical Gerontologist*.

[23] Greenberg S. A. (2012). The geriatric depression scale (GDS). *Best Practices in Nursing Care to Older Adults*.

[24] Patel V., Tiwari D., Shah V., Patel M., Raja H., Patel D. (2018). Prevalence and predictors of abuse in elderly patients with depression at a tertiary care centre in Saurashtra, India. *Indian Journal of Psychological Medicine*.

[25] Ranjan S., Bhattarai A., Dutta M. (2013). Prevalence of depression among elderly people living in old age home in the capital city Kathmandu. *Health Renaissance*.

[26] Chalise H., Basnet M. (2017). Abuse of older adults residing in the community of Nepal. *Journal of Gerontology and Geriatric Research*.

[27] Shankardass M. K., Rajan S. I. (2018). *Abuse and Neglect of the Elderly in India*.

[28] Yadav U. N., Tamang M. K., Paudel G. (2018). The time has come to eliminate the gaps in the under-recognized burden of elder mistreatment: a community-based, cross-sectional study from rural eastern Nepal. *PloS one*.

[29] Anand A. (2016). Exploring the role of socioeconomic factors in abuse and neglect of elderly population in Maharashtra, India. *Journal of Geriatric Mental Health*.

[30] Udayar S. E., Devika P., Konduru R. K., Patil S. D. (2014). A study of economic dependency and its relation to depression among elderly people in rural area of Chittoor district, Andhra Pradesh. *International Journal of Health Sciences and Research*.

[31] Akhtar H., Khan A. M., Vaidhyanathan K. V., Chhabra P., Kannan A. (2013). Socio-demographic predictors of depression among the elderly patients attending out patient departments of a tertiary hospital in North India. *International Journal of Preventive Medicine*.

[32] Manandhar K., Risal A., Shrestha O. (2019). Prevalence of geriatric depression in the Kavre district, Nepal: findings from a cross sectional community survey. *BMC Psychiatry*.

[33] Yadav U. N., Thapa T. B., Mistry S. K., Pokhrel R., Harris M. F. (2020). Socio-demographic characteristics, lifestyle factors, multi-morbid conditions and depressive symptoms among Nepalese older adults. *BMC Psychiatry*.

[34] Malhotra R., Chan A., Ostbye T. (2010). Prevalence and correlates of clinically significant depressive symptoms among elderly people in Sri Lanka: findings from a national survey. *International Psychogeriatrics*.

[35] Novelo M., von Gunten A., Gomes Jardim G. B., Spanemberg L., Argimon I. I. . L., Nogueira E. L. (2018). Effects of childhood multiple maltreatment experiences on depression of socioeconomic disadvantaged elderly in Brazil. *Child Abuse & Neglect*.

[36] Kraaij V., de Wilde E. J. (2001). Negative life events and depressive symptoms in the elderly: a life span perspective. *Aging & Mental Health*.

[37] Santos A. J., Nunes B., Kislaya I., Gil A. P., Ribeiro O. (2021). Exploring the Correlates to Depression in Elder Abuse Victims: Abusive Experience or Individual Characteristics?. *Journal of Interpersonal Violence*.

[38] Roh S., Burnette C. E., Lee K. H., Lee Y.-S., Easton S. D., Lawler M. J. (2015). Risk and protective factors for depressive symptoms among American Indian older adults: adverse childhood experiences and social support. *Aging & Mental Health*.

[39] Khalili Z., Taghadosi M., Heravi-Karimooi M., Sadrollahi A., Gilasi H. (2016). Assessment of the associations of depression with elder abuse among the elderly in Kashan city, Iran. *Iranian Journal of Ageing*.

[40] Hafemeister T. L. (2003). *Financial Abuse of the Elderly in Domestic Settings Washington District of Columbia*.

